# Notes and News

**Published:** 1903-08-15

**Authors:** 


### NOTES AND NEWS.

The King has granted a Royal Charter of Incorporation to the National Hospital for the Paralysed and Epileptic.

There is still a good deal of small-pox prevalent at Cambridge. Sixty-one cases are reported in hospital, and a total of 78 under treatment.

Sir William Ramsay received the honorary degree of Doctor of Medicine on the occasion of the celebration of the hundredth anniversary of the foundation of the University of Heidelberg. Several other distinguished English men and women received honorary degrees.

Colonel Davies Evan, Lord-Lieutenant of Cardiganshire, has provided a site upon easy terms for a sanatorium for the West Wales branch of the National Association for the Prevention of Consumption. Estimates are to be obtained for a movable sanatorium for 20 patients with annexes for paying patients. Sir John Williams, who has retired to Wales, is associating himself with the movement.

In the report of the Royal Commission on Alien Immigration, just laid before the House of Commons, the salient point is practically the stress laid upon the overcrowding caused by the alien population of the metropolis. From our standpoint the sanitary objections are amongst the most important that can be raised in favour of a check of some kind being introduced. It is impossible to improve the general housing of the London poor when every new locality becomes crowded by those who are thrust out from quarters where the alien population chooses to congregate. The foreign population exist under conditions even worse than our own, and the sanitary authorities have little chance of establishing even the most primitive standard of cleanliness whilst the hording together of the undesirable is permitted as at present

Dr. Canton has been appointed Harveian Orator for 1904.

There were 17 cases of the Plague reported from Mauritius last week, with 14 fatalities.

The proceeds of the ball at the Royal Albert Hall in aid of the London Hospital amounted to £2,656.

The German Association of Scientists and Physicians will hold their annual meeting at Cassel from September 20th to 26th.

The Bradshaw Lecture in connection with the Royal College of Physicians will be delivered this year by Dr. Trevelyan on November 5th. The subject will be Tuberculosis of the Nervous System.

An International Congress on Tuberculosis will be held in Paris in September and October in next year. The Congress will be divided into three sections — Medical, Social, and Technical. The Museum will be arranged in connection with the Congress, of which Professor Broardel will be President.

The Board of Agriculture have made arrangements with the National Physical Laboratory for the verification of milk-testing apparatus, which will receive the "hall mark" of the laboratory, after examination for a small fee. Copies of the regulations for examination and testing can be obtained from the Director, Bushey House, Teddington.

Coming at the present time the report of the Committee appointed by the Royal College of Physicians to consider the safety and value of anti-typhoid inocculation is of special interest. Briefly the Committee consider that susceptibility and mortality are decreased by its adoption, but they recommend great caution at the time of inocculation, as they regard susceptibility to infection to be temporarily increased.

The Hop-picking Mission Committee point out that in a short time Mid and West Kent will be invaded by some 40,000 or more hop-pickers, for the most part from London, and chiefly of the class that live from hand to mouth. There is generally a considerable amount of sickness amongst them, and this the Mission tries to combat by sending trained nurses to work from different centres, opening small hospitals for the children and dispensaries for first aid. In addition they provide and work coffee-stalls and barrows, and open reading and writing rooms in the evenings and on Sundays. Illustrated papers, magazines, etc., are distributed in the tents and huts where the pickers are housed. The Mission is asked to supply over forty experienced lady workers and nurses this year, and as their work is dependent upon public subscriptions, they appeal for money, nursing requisites, and literature; £5 pays all expenses of a nurse or worker, whose services are voluntary. Subscriptions and parcels may be sent to the Rev. F. Oliphant, Teston Rectory, Maidstone.

